# Exploring Neural Evidence of Attention in Classroom Environments: A Scoping Review

**DOI:** 10.3390/brainsci15080860

**Published:** 2025-08-13

**Authors:** Hang Zeng, Xinmei Huang, Yelin Liu, Xiaojing Gu

**Affiliations:** 1 Center for Educational Science and Technology, Beijing Normal University, Zhuhai 519087, China; 2 Faculty of Education, Beijing Normal University, Beijing 100875, China

**Keywords:** classroom, attention, neural mechanisms, educational neuroscience, EEG

## Abstract

Classroom attention is a fundamental cognitive function that is crucial to effective learning and significantly influences academic performance. Recent advances in investigating neural correlates of attention in classroom environments provide insights into underlying neural mechanisms and potentially enhance educational outcomes. This paper presents a scoping review of empirical studies investigating neural activities associated with students’ attention in classroom environments. Based on the 16 studies that we included after systematically searching, five main objectives were identified: (i) examination of neural markers of student attention in classroom environments, (ii) comparison of different learning environments, (iii) comparison of different classroom activities, (iv) data quality examination, and (v) student attention improvement. All selected studies used electroencephalogram (EEG) recording to measure neural activities, primarily using NeuroSky and Emotiv EPOC devices. Researchers measured classroom attention through brain-to-brain synchrony or frequency power. While differences in neural activity across classroom activities were noted, further investigation is needed for consistent results. Most studies focused on university students and had limited sample sizes, though they covered diverse study domains. Overall, while some preliminary results have been identified, there are several concerns regarding the neural measurements of attention used, contradictory findings, lack of verification, and limited sample sizes and techniques. Further studies are recommended to extend our understanding of neural evidence of attention in classroom environments.

## 1. Introduction

### 1.1. Classroom Attention and Its Measurements

Attention is a fundamental cognitive function that enables individuals to process pertinent information from their environment. It plays a crucial role in various aspects of daily life, influencing our ability to function effectively and efficiently. In the context of education, attention is a vital component of the learning process, significantly affecting academic performance and overall educational outcomes [1,2]. Despite its importance, maintaining or regulating students’ attention in the classroom poses significant challenges for educators due to factors related to student behavior, instructional methods, and environmental influences [3,4]. For instance, students often experience attention lapses during lectures, partly due to the limited attentional span of students, and partly due to the passive nature of traditional lecture formats [5,6,7]. Moreover, the use of devices such as mobile phones in the classroom can interfere with attention and negatively impact academic performance [8]. Additionally, classrooms frequently contain numerous visual stimuli, such as posters and artwork, which can distract students from the lesson [9]. Consequently, measuring or monitoring classroom attention and potentially enhancing students’ attention in classroom environments is a crucial research topic.

Previous studies have utilized subjective measures, such as self-reports and questionnaires [10] or behavioral observations [11], to assess students’ attention and engagement in the classroom. However, these methods are prone to subjective bias and may not provide objective and accurate measurements. More recent techniques, such as eye-tracking [12,13,14] and facial and body feature analysis [15,16,17,18,19], offer improved methods for measuring classroom attention.

In addition to these behavioral indicators, investigating the neural activities associated with students’ attention in classroom environments has garnered significant interest [20,21,22]. This approach can help elucidate the underlying neural mechanisms of attention in a natural setting. Neural investigations can also detect covert attention shifts, which are not accompanied by observable behaviors, thereby uncovering subtle attentional processes that might be overlooked by behavioral education research [23,24].

### 1.2. Investigating Neural Activities in Classroom Environments

Prior to the development of real-world and educational neuroscience, cognitive neuroscience research predominantly involved neuroimaging studies conducted under strictly controlled laboratory conditions. These studies aimed to isolate and investigate basic neural mechanisms of cognition, with the assumption that findings would generalize to natural environments such as classrooms [25]. However, the extent to which these findings reflect the complexity of cognitive processing in natural settings remains largely unknown. Many researchers argue that a significant gap exists between results obtained in controlled laboratory environments and the dynamic, complex, and multisensory nature of real-world contexts [26,27,28].

In response, researchers have begun to bridge this gap by conducting experiments in more naturalistic settings, such as classrooms. According to Dikker et al. [29], classrooms provide an ideal starting point for real-world neuroscience, offering a practically important and ecologically valid context while maintaining a semi-controlled environment governed by teacher-led activities. Their study was also one of the earliest to investigate classroom engagement and social dynamics using neural techniques. Since then, an increasing number of researchers have focused on exploring the neural activities related to classroom attention.

### 1.3. Rationale for the Review

With the advancement of educational neuroscience, numerous reviews have been conducted to map and evaluate research in this emerging field. Xu and Zhong [30] reviewed the use of portable electroencephalogram (EEG) technology (PEEGT) in educational research, revealing that PEEGT was primarily employed to evaluate learners’ attention and meditation. They also noted the lack of studies in naturalistic classroom settings, which has changed since their review. Later, Janssen et al. [31] explored the growing use of mobile neuroimaging technologies in the field of educational neuroscience, focusing on both the potential benefits and the challenges that these technologies bring to the study of learning in real-world settings. Importantly, they proposed a cyclical research model that bridges lab-based neuroscience with classroom-based studies, offering a framework for integrating findings across contexts, particularly in domains such as attention research. Research by Tenório et al. [32] systematically reviewed brain-imaging techniques used in educational technologies, offering a comprehensive overview of the brain-imaging methods, devices, educational levels, study domains, and outcomes of empirical studies. However, Tenório et al. [32] emphasized technology-enhanced learning environments, such as Massive Open Online Courses (MOOCs) and virtual learning environments, over traditional classroom settings, which remain the predominant learning environment.

More recently, several reviews have emerged that touch on neural evidence in the classroom, such as interpersonal educational neuroscience [33] and hyperscanning studies in learning [34]. They primarily emphasize instructor–learner or peer interactions using hyperscanning approaches, rather than the neural correlates of attention. Furthermore, Nouri [35] offers a broad statistical overview of educational neurotechnology, encompassing 450 studies but without engaging deeply with specific neural processes like attention.

Therefore, despite the increasing neural evidence of students’ attention in classroom environments, no review has yet synthesized this evidence to provide a comprehensive understanding of the field. From an educator’s perspective, neural evidence can offer objective measures of attention, complementing traditional behavioral assessments and yielding a more thorough understanding of student attentional engagement, which could inform the design of classroom activities that promote sustained attention. For example, several studies have compared attentional states across different classroom activities [29,36,37,38,39]. Furthermore, analyzing the temporal patterns of attention across classroom episodes can reveal when and why attention lapses occur, providing actionable insights to enhance teaching effectiveness and learning outcomes. From a cognitive neuroscientist’s perspective, studying attention in naturalistic settings like classrooms not only adds ecological validity to cognitive neuroscience research, but also provides a rich context for examining how attention is influenced by various factors, including social interactions, emotional states, and environmental variables. As Stangl et al. [40] stated, insights and potentially unexpected findings from real-world studies will provide a foundation for developing new theories and models of neuroscience.

### 1.4. The Current Study

The purpose of this paper is to provide a scoping review of existing empirical studies of the neural activities of students’ attention in classroom environments. Given that this research field is relatively new and under-explored, and considering our broad and exploratory research aim, we opted to perform a scoping review [41]. This approach also helps identify gaps in the current knowledge base, informing future research agendas.

In this review, we define attention as closely related cognitive processes involving selective concentration on relevant information while filtering out distractions, and maintaining focus over an extended period. These two dimensions are particularly relevant in dynamic classroom environments, where students are constantly required to stay focused and manage multiple sources of information. Additionally, through our initial review of the literature, we observed that many studies use the term engagement to refer to similar cognitive processes as described above. Therefore, we include engagement as a search keyword in this review and define it in a manner analogous to attention, unless otherwise stated.

Specifically, this scoping review aims to answer the following five research questions:

RQ1: What objectives and outcomes are reported in the included studies?

RQ2: What techniques and devices are used in the included studies?

RQ3: What neural and behavioral measurements of attention are reported in the included studies?

RQ4: What differences in neural activity are observed across various classroom activities in the included studies?

RQ5: What are the educational levels, disciplinary domains, durations, and sample sizes of the included studies?

## 2. Methods

This scoping review has been registered with the Open Science Framework (OSF). The protocol for this review was developed according to Peters et al. [42] and the Preferred Reporting Items for Systematic Reviews and Meta-Analyses (PRISMA)–extension for scoping review [43] and can be accessed at https://osf.io/z82su, accessed on 1 July 2025. Below, we report the inclusion and exclusion criteria, the literature search, the literature selection, and the data coding and extraction [44].

### 2.1. Inclusion and Exclusion Criteria

We established clear inclusion and exclusion criteria to guide the selection of relevant literature, as outlined in Table 1. Eligible sources included original research studies such as peer-reviewed journal articles, dissertations, and conference papers. We excluded non-original research publications, including literature reviews, meta-analyses, editorials, book reviews, and reports. The focus of the literature had to be on investigating neural activity, neural mechanisms, or brain signals of attention and engagement within face-to-face classroom settings. Studies that focused solely on behavioral aspects without a neural component, those centered exclusively on online learning or e-learning environments, which have been covered by Tenório et al. [32], and studies unrelated to attention were excluded. We also excluded studies that linked neural activity solely to learning outcomes (e.g., performance or memory) without directly measuring attention or engagement. Such outcomes are influenced by multiple factors beyond attention, and including these studies would have introduced conceptual variability inconsistent with our review’s objective.

Studies conducted in any country were eligible, but only articles published in English were included. We considered studies published between 2001 and 2024, aligning with Tenório et al. [32], who identified 2001 as the starting point for relevant research in brain imaging and educational technologies. Since investigating neural activity in educational settings is a relatively new research topic, research prior to 2001 was unlikely to be relevant. Studies involving healthy participants of all age groups and covering various educational levels, across all disciplinary fields, were considered.

### 2.2. Literature Search

We began by conducting an electronic search across key databases relevant to neuroscience, psychology, and education. These included Web of Science, EBSCOhost (covering Academic Search Ultimate, APA PsycTests, Psychology and Behavioral Sciences Collection, ERIC, MEDLINE, and OpenDissertations), and ProQuest (including APA PsycInfo, ProQuest Dissertations & Theses Global, Psychology Database, Education Database, and APA PsycArticles^®^). The search was carried out on 14 May 2024. For Web of Science, we used the following search string: ((TS = (attention or engagement) AND TS = (classroom or school or lecture)) AND TS = (neural or neuroscience or brain*). Additional filters for publication date, language, and document type were also applied. To ensure that the search strategy was appropriately balanced, we refined the search string through multiple trial runs, aiming to avoid both excessive specificity, which could exclude relevant studies, and excessive breadth, which could yield an unmanageable volume of results. While the core search terms remained consistent across databases, the syntax was adjusted to fit the specific requirements of each platform.

### 2.3. Literature Selection

**Figure 1:**
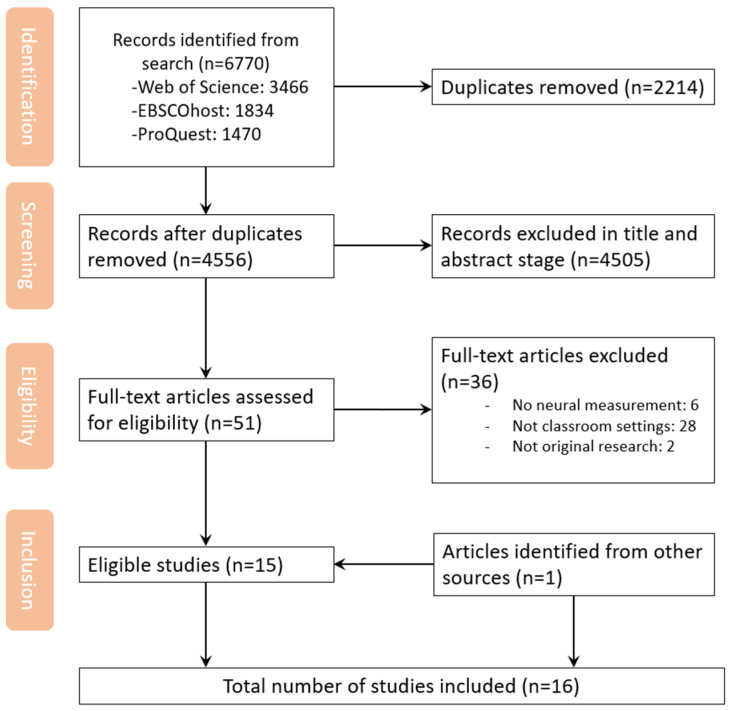
Study selection flow diagram.

Two reviewers independently screened the studies for eligibility using a hierarchical approach. All identified articles were organized and managed using Zotero 6.0.36. In the first stage, titles and abstracts retrieved from the database searches were screened to identify potentially relevant studies. In the second stage, the reviewers assessed the full-text articles, excluding those that did not meet the predefined eligibility criteria. In the third stage, we conducted both backward and forward reference searches (snowballing) to ensure comprehensive coverage. For the backward search, we examined the reference lists of included articles to identify additional studies that may not have appeared in the original database searches but were potentially relevant. In the forward search, we used Google Scholar to locate articles that had cited the included studies. We also reviewed the reference lists of prior relevant reviews to capture any further eligible literature. The overall study selection process is illustrated in the PRISMA flowchart (see Figure 1). Inter-rater reliability for the screening process was calculated using Cohen’s Kappa, resulting in κ = 0.85, indicating a high level of agreement. Any disagreements were resolved through discussion until a consensus was reached.

### 2.4. Data Coding and Extraction

Two independent raters coded the included studies using a structured coding form (see Table 2). The information extracted included publication characteristics as well as data relevant to each research question. The inter-rater reliability, assessed using Krippendorff’s alpha, was 0.80, reflecting strong agreement between coders. As before, discrepancies were discussed and resolved through consensus.

## 3. Results

### 3.1. Search Results

The initial database search yielded 6770 articles, which was reduced to 4556 after the removal of duplicates. Following title and abstract screening, 4505 articles were excluded for not meeting the eligibility criteria, leaving 51 articles for full-text review. Of these, 15 met the inclusion criteria. An additional eligible article was identified through other sources. In total, 16 articles were included in this scoping review (see Figure 1).

Examples of articles that initially appeared to meet the inclusion criteria but were ultimately excluded may illustrate how the inclusion criteria were applied. Both Lin and Chen [45] and Poulsen et al. [46] investigated neural correlates of students’ attention in a classroom environment. However, their studies focused solely on video or movie watching without teacher instruction or supervision. We determined that these studies did not involve a classical classroom environment typically characterized by a teacher leading several classroom activities, resulting in more dynamic, interactive environments. These two articles were therefore excluded.

### 3.2. Description of Included Studies

The included studies were published between 2014 and 2024 (see Table 3), with 15 of the 16 studies published from 2017 onward, reflecting the emerging nature of this research area. Across all studies, the total number of participants whose neural activity was recorded amounted to 255. All studies appeared in peer-reviewed journals. Geographically, seven studies were conducted in the United States, five in mainland China or Taiwan, and the remaining four in Canada, India, Israel, and Spain.

### 3.3. What Objectives and Outcomes Are Reported in the Included Studies?

**Figure 2:**
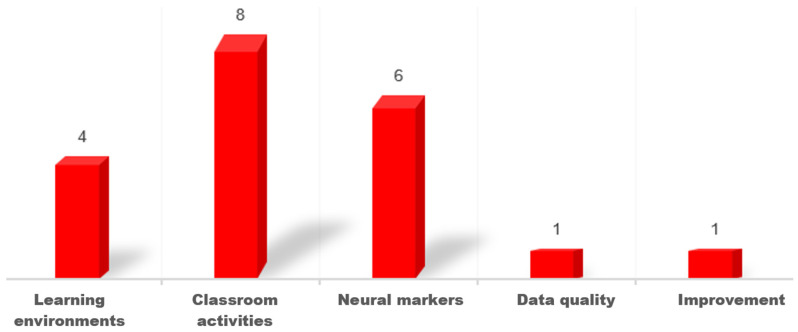
Research objectives of the selected papers.

After analyzing the 16 papers, five types of objectives were classified, and some papers covered more than one objective (see Figure 2).

#### 3.3.1. Examining Neural Markers of Students’ Attention in Classroom Environments

Six of the selected papers aimed to identify neural markers that can capture or predict students’ attentional states in classroom environments.

Four of them focused on brain-to-brain synchrony [29,36,48,49]. Brain-to-brain synchrony (also called inter-brain synchrony) refers to the temporal alignment of neural activity between two or more individuals, typically measured using tools like EEG or functional near-infrared spectroscopy (fNIRS) while participants are engaged in a shared activity such as communication or learning. Specifically, Dikker et al. [29] examined how brain-to-brain synchrony among a group of high school students in a classroom setting relates to their engagement and social dynamics, finding that students’ brain-to-brain synchrony predicts their classroom engagement and social dynamics, such as how much they like the teacher and each other. They suggest that, when students are jointly focused on the same stimulus or actively collaborating, their brain signals tend to become more synchronized, indicating a shared cognitive state (shared attention). This makes brain-to-brain synchrony particularly useful in naturalistic educational settings, where learning is inherently social. Compared to traditional metrics such as frequency band power—which reflects individual-level neural activation—brain-to-brain synchrony offers a dyadic or group-level perspective on attention and engagement, capturing interpersonal dynamics that individual metrics cannot.

Later, based on such findings, and the fact that attention increases retention, Bevilacqua et al. [36] further tested the hypothesis that a student’s neural synchrony to the rest of the group or with the teacher predicts their retention of the content. They replicated and extended previous findings by demonstrating that teacher–student synchrony varied with student engagement and teacher likability. However, they found no significant relationship between student-to-group brain synchrony and lesson content retention. The researchers suggested that this null result might be attributed to limitations such as a small sample size and the use of commercial-grade EEG devices in classroom settings, which may have led to a low signal-to-noise ratio.

To address these limitations, the research team continued testing the hypothesis with a larger sample and research-grade EEG equipment [49]. They found that alpha-band synchrony (measured by phase alignment) among students significantly predicted both immediate and delayed posttest performance, after controlling for pretest scores, a factor that was not considered in the previous study. This study is among the first to demonstrate the relation between phase-based alpha band inter-brain synchrony and learning. The authors argued that, when task engagement increases, students’ alpha oscillations are attenuated but become more phase-entrained with the external stimulus (e.g., the lecture), leading to higher brain-to-brain synchrony across students.

Similarly, Chen et al. [48] proposed an inter-brain attention coupling analysis framework to detect learning-related attention in primary school students and found a positive correlation between students’ inter-brain attention coupling and class-average attention dynamics during lectures and academic performance. Unlike the three studies mentioned above, Chen et al. [48] first averaged the brain signals across all participants to create a single “class-average” attention time series, then computed the correlation between each individual’s neural data and this group-average signal. In contrast, Dikker et al. [29] and related work first computed pairwise coherence between the brain signals of each student–student pair, then averaged all possible pairwise combinations between one participant and the rest of the group to derive a “student–group synchrony” metric. Because Chen et al.’s method relies on a single aggregated reference signal [48], it is inherently more sensitive to global class dynamics—capturing moments when the entire group collectively shifts attention. By correlating each child’s brain activity with that global pattern, their approach highlights how closely each student tracks the class’s overall attentional state. On the other hand, the pairwise-coherence approach used by Dikker et al. [29] emphasizes bi-directional, dyadic alignment and can identify subgroups or individual pairs that may be more (or less) synchronized than the class as a whole.

Apart from brain-to-brain synchrony, machine learning and traditional spectral power analysis were also used. Power analysis of EEG frequency bands is a widely used method in neuroscience to assess cognitive states. EEG signals are decomposed into distinct frequency bands—most commonly delta (1–4 Hz), theta (4–8 Hz), alpha (8–13 Hz), beta (13–30 Hz), and gamma (>30 Hz)—each associated with different neural and psychological processes. In the context of attention, decreased alpha power, especially in the parietal and occipital regions, is commonly interpreted as an indicator of increased attentional focus, reflecting reduced cortical idling. Other frequency bands have also been shown to indicate attentional allocation or cognitive engagement states. Ko et al. [53] examined changes in EEG spectral power related to performance on a sustained attention task in a real classroom, identifying variations in EEG dynamics, such as increased delta and theta power in the occipital region and decreased delta, theta, and alpha power in the frontal region, associated with longer response times (lower attention). In their study, while students were listening to a lecture, they were instructed to respond to a visual target that occasionally appeared on the classroom screen by pressing a corresponding button on their smartphones. Although this task was designed to probe students’ attention in the classroom, it introduced an unnatural element that complicates the interpretation of the findings. For example, if a student was focused on the lecturer rather than the classroom screen, their reaction time might be delayed, but not because they were inattentive.

Dhindsa et al. [50] used EEG and machine learning to detect mind wandering during live lectures, finding that neural correlates of mind wandering were highly individualized, despite some group-level similarities. Specifically, while some common EEG features (such as changes in alpha power) emerged across participants, the machine learning models trained on one individual’s data did not generalize well to others. This suggests that the neural signatures of attentional lapses are subject to considerable inter-individual variability, highlighting the importance of personalized approaches in real-time attention detection.

#### 3.3.2. Comparison of Different Learning Environments on Neural Attention Levels

Four of sixteen papers compared neural attention levels across different learning environments, considering aspects of modalities and timing. Three of these studies compared the neurobiological correlates of attention (measured by EEG) between online learning and traditional classroom learning environments [47,51,52]. Aggarwal et al. [47] found higher and more sustained attention levels in MOOC/e-learning than in the traditional classroom environment. However, Horowitz-Kraus et al. [51] found that classroom learning resulted in higher comprehension levels and greater neural synchrony between teacher and students, as well as among the students themselves, compared to online learning. Juárez-Varón et al. [52] demonstrated that students who attended class in person had higher levels of emotional arousal, interest, stress, and engagement (emotional) compared to those who attended online, while focused attention levels were similar between the two groups. In their study, engagement encompasses a broader set of affective and behavioral responses, while attention refers to the cognitive process of selectively concentrating on specific information while ignoring other stimuli.

One study aimed to assess the effects of different class times during the day, focusing on the temporal aspect of learning environments [37]. Researchers recorded brain activity from 22 students across two schools during early morning, mid-morning, and afternoon biology classes. They focused on alpha-band EEG activity, which is known to inversely correlate with attention. Results showed lower attention and worse performance in the early morning classes compared to the mid-morning classes.

#### 3.3.3. Comparison of Different Classroom Activities on Student Attention

Eight of sixteen papers compared the neural activities of attention during different classroom activities (see Table 4). Classroom activities include lectures, video-watching, group discussion/work, individual work, different polling strategies, and mindfulness. While all eight studies involved lectures, five out of eight studies additionally examined video-watching and/or student-led activities such as group discussion/work [29,36,37,38,39]. Especially, three out of eight studies focused on different polling strategies conducted by the same group of researchers [55,56,57]. The attentional neural correlates of different classroom activities are further discussed below.

#### 3.3.4. Data Quality Examination in Classroom Environments

Despite the significance of this research topic, Xu et al. [39] was the only study to examine the feasibility of collecting high-quality EEG data in increasingly naturalistic settings, ranging from a lab-based paradigm to a semi-naturalistic classroom setting. They first conducted an experiment in a controlled, lab-like setting using a wired EEG system, followed by an experiment in real classroom environments using mobile EEG equipment. The study concluded that it is feasible to collect high-quality EEG data from young children in both lab-based and classroom-based settings, although there was an increase in data loss and noise in the classroom setting.

#### 3.3.5. Using Biofeedback Technique to Improve Student Attention

Kosmyna and Maes [54] was the only study among the included papers that aimed at improving students’ performance in the classroom by providing feedback on attention. They developed a prototype of a wearable system called AttentivU that uses an EEG headband to measure engagement in real-time and a scarf that provides haptic feedback when engagement drops. They tested the effectiveness of this system in two studies, one with video lectures and one with live lectures (classroom setting), comparing three feedback conditions: biofeedback, random feedback, and no feedback. The results showed that the device increased user engagement and improved learning performance when providing biofeedback on drops in engagement. Another study demonstrating the feasibility of using neurofeedback techniques to regulate students’ attention in a classroom comes from Janssen and van Atteveldt [58]. Although the primary aim of the research was to enhance the effectiveness of a growth mindset intervention, researchers designed a session dedicated to providing students with direct neurofeedback on their brain activity, thereby reinforcing the concept of brain plasticity in learning. Specifically, theta/beta ratio neurofeedback, a well-established neural marker of attention, was implemented with over 200 high school students in real classroom settings. The study provides evidence that students can, in fact, learn to modulate neural indicators of attention in a classroom.

### 3.4. What Techniques and Devices Are Used in the Included Studies?

**Figure 3:**
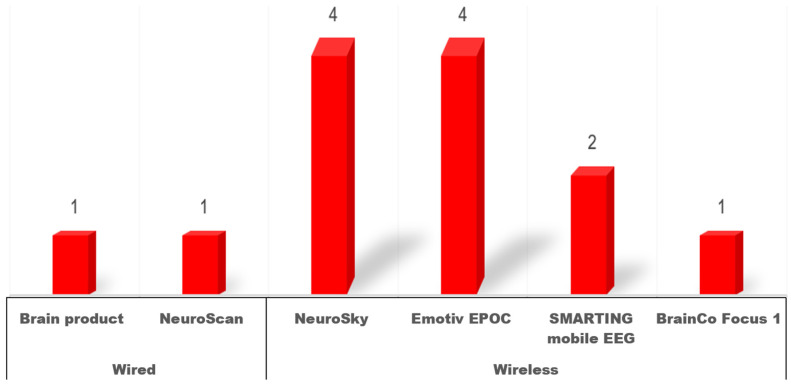
Information on EEG devices used in the included studies.

All of the included studies employed EEG recording techniques to measure neural activity (see Table 3 and Figure 3). However, three of these studies did not specify the devices used. Two studies utilized wired EEG devices: NeuroScan with 32 channels [53] and Brain Products with 32 channels ([39] Study 1). The remaining studies employed mobile EEG devices. The most frequently used mobile devices were NeuroSky, with 1 channel (four studies: [47,48,56,57]), and the Emotiv EPOC device with 14 channels (four studies: [29,36,37,52]). Additionally, two studies used the SMARTING mobile EEG system with 24 channels ([38,39] Study 2), and one study used BrainCo Focus 1 with 1 channel [54].

These variations in EEG device types highlight the need for a standardized approach to device selection in this field. Wired systems with a higher number of channels offer superior spatial resolution and signal-to-noise ratio but are less practical for real-world classroom use due to their limited mobility and complex setup requirements. In contrast, mobile EEG systems—particularly low-density options like NeuroSky and BrainCo—prioritize ease of use and comfort, enabling researchers to collect data in naturalistic classroom settings with minimal disruption. Mid-density mobile systems such as Emotiv EPOC and SMARTING offer a compromise between signal resolution and wearability. Their wireless design and moderate channel count support more detailed neural recordings while remaining suitable for real-time deployment in classrooms.

### 3.5. What Neural and Behavioral Measurements of Attention Are Reported in the Included Studies?

Except for two studies that did not report the neural measurements used in their study, we categorized the neural measurements into two types: brain-to-brain synchrony and power analysis of frequency bands (see Table 5).

Five studies used brain-to-brain synchrony to measure students’ classroom attention or engagement level [29,36,48,49,51]. The synchrony was measured either between a student and their peers or between a student and their teacher. Dikker et al. [29] and Bevilacqua et al. [36] also included behavioral measurements of attention to establish or verify the positive link between brain-to-brain synchrony and attention/engagement states. There are some variations in how brain-to-brain synchrony was calculated. Dikker et al. [29] and Bevilacqua et al. [36] used EEG signals in the frequency range between 1 and 20 Hz at F3, F4, P7, P8, O1, and O2 sites. Davidesco et al. [49] focused on the phase-based alpha band synchrony from 32 channels. Horowitz-Kraus et al. [51] tested neural data from alpha (8–12 Hz) and beta (12–30 Hz) frequency bands at frontal electrodes (AF3, AF4, F4, F3, F8, and F7). Targeting the “class-average” attention, Chen et al. [48] calculated synchrony using attention level data provided by the Attention Meter algorithm from NeuroSky, which only uses one frontal electrode (see http://neurosky.com/biosensors/eeg-sensor/algorithms/, accessed on 1 July 2025). Given the differences in frequency bands and electrode sites across studies, caution may be warranted when interpreting brain-to-brain synchrony. It remains an open question whether synchrony measures derived from varying frequency ranges and electrode locations are directly comparable.

The remaining 10 studies used power analysis of frequency bands to measure attentional levels. Among them, two studies used self-trained classification models based on eight frequency bands [47] or four frequency bands [50] to detect mind wandering or measure attentional states in classroom environments. Neural data were labelled as attentive or inattentive according to the behavioral measurements of attention. However, such an approach might be less ideal to identify covert attention, which often eludes behavioral measures.

Three studies used attention level data provided by the patented algorithm from NeuroSky [48,56,57]. This algorithm indicates the intensity of mental “focus” or “attention”, with scores ranging from 0 to 100, where higher scores represent greater attentiveness. EEG power spectrums (alpha, beta frequency, etc.) are employed to calculate these attentional scores [30]. Although an attention score may be easy for users to interpret, it should be approached with caution, as the exact algorithms or methods used to compute these scores are often not fully disclosed.

Five studies used classical power spectrum analysis pipelines to measure attentional neural activities, with alpha power being the most frequently used indicator due to its well-established negative correlation with attentional levels. Xu et al. [39] utilized alpha power (7.5–12 Hz) at electrodes Pz, POz, O1, and O2 as the neural indicator of attention. Dikker et al. [37] assessed alpha power (7–14 Hz) at occipital electrodes and also recorded students’ self-reported focus scores. Grammer et al. [38] also collected alpha power (7.5–12.5 Hz) and observational data of attention. Ko et al. [53] used a sustained attention task to probe the neural correlates of reaction times, revealing that decreased alpha power (8–14 Hz) in frontal and temporal regions was associated with prolonged reaction times, indicating reduced attentiveness.

Other frequency bands, such as beta, theta, and delta, have been less frequently examined in relation to attention. Ko et al. [53] reported that prolonged response times were preceded by an increase in delta and theta powers over the occipital region and a decrease in beta power over the occipital and temporal regions. Kosmyna et al. [54] utilized index E = β/(α + θ), where α (7–11 Hz), β (11–20 Hz), and θ (4–7 Hz), to measure student attention.

### 3.6. What Differences in Neural Activity Are Observed Across Various Classroom Activities in the Included Studies?

The included studies examined various classroom activities, as summarized above. Here, we analyze the differences in neural activity across these activities.

Firstly, Dikker et al. [37], Grammer et al. [38], and Xu et al. [39] used alpha power to measure student attention and compared differences in alpha power during different teaching activities such as lectures, video-watching, and student-led activities. Dikker et al. [37] found that alpha power was lower during video-watching compared to lectures, while Grammer et al. [38] found that alpha power was higher during video-watching compared to lectures and student-led activities. When comparing teacher-led and student-led activities, Grammer et al. [38] found that alpha power was lower during teacher-led activities, while Xu et al. [39] reported no significant difference between the two types of activities. Xu et al. [39] suggested that this discrepancy could reflect developmental differences or variations in classroom experience, as elementary school activities are generally more interactive compared to high school and college classrooms. We further discuss the contradictory findings in the Discussion section.

Using brain-to-brain synchrony as an index of class engagement, Dikker et al. [29] and Bevilacqua et al. [36] found that inter-brain synchrony was higher during video sessions compared to lectures, aligning with findings from Dikker et al. [37]. Additionally, Dikker et al. [29] found that inter-brain synchrony was higher during group discussions compared to lectures, with no significant difference between video sessions and group discussions.

Lastly, Sun and colleagues are interested in how different polling strategies affect students’ attentional brain waves. In their earlier study, they found that students’ brainwave data related to attention increased during polling activities compared to lectures in general [55]. However, later studies did not identify a consistent pattern across the three participants they collected, suggesting that different students may benefit from various instructional methods and activities to maintain high attention levels [56,57].

### 3.7. What Are the Educational Levels, Disciplinary Domains, Durations, and Sample Sizes of the Included Studies?

Regarding the participant population, the largest research group consisted of university students, with 10 studies encompassing both undergraduate and graduate students. Four studies focused on high school students, three on primary school students, and one on kindergarten children (see Table 6).

As shown in Table 6, a diversity of study domains was covered in the included studies. The most frequently investigated domain was Biology (4 out of 16 studies). Other disciplines explored included Brain sciences, Educational neuroscience, Machine learning, and VR.

The duration was calculated by multiplying the duration of each session by the number of sessions. Sun [55] reported only the times of session, and Dikker et al. [37] used data from Dikker et al. [29] and Bevilacqua et al. [36], so these two studies were excluded from the analyses. Study durations varied widely, ranging from less than 20 min to 600 min, with an average duration of approximately 208 min and a standard deviation of 196 min. Five out of fourteen studies had durations of 60 min or less, six studies recorded durations between 61 and 300 min, and the remaining three studies recorded durations between 301 and 600 min (see Table 6).

Sample sizes with neural data collected were analyzed. The average sample size is around 16, with a standard deviation of 8.6. Three studies recruited fewer than 10 participants, with the smallest sample size three 3 students. Eight studies had sample sizes ranging from 11 to 20. Only five studies recruited more than 20 participants, with the largest sample size being 32 (see Table 6).

## 4. Discussion

The current study explored the neural evidence of attention in classroom environments through a scoping review with 16 studies. While 16 studies might seem limited, it is important to note that research on neural evidence of attention in classroom environments is an emerging field. Fifteen out of the sixteen studies included were published since 2017, indicating the recency of and growing interest in this area. By highlighting both the strengths and limitations of the existing research, this review aims to guide future studies in expanding and refining this important area of inquiry. This scoping review provides a timely synthesis of an emerging field that investigates neural evidence of attention in classroom settings, offering researchers a comprehensive overview of current methodologies, challenges, and directions for future work. By highlighting both the strengths and limitations of the existing research, this review aims to advance theoretical understanding and guide future studies in expanding and refining this important area of inquiry.

Several meaningful findings can be summarized. Firstly, it is feasible to measure the neural activity of students in classroom environments using EEG, although the issue of data loss must be considered. Secondly, brain-to-brain synchrony and power oscillations, especially alpha power oscillations, can be utilized as effective neural markers to identify students’ attention or engagement levels in classroom settings (but see the below discussion for potential limitations). While these preliminary results are promising, several concerns in the research field need to be addressed.

### 4.1. Limitations Regarding Neural Measurements of Attention

Five studies used brain-to-brain synchrony as the neural measurement of attention in classroom settings. However, some of the studies used data of frequency range between 1 and 20 Hz from frontal, parietal, and occipital electrodes [29,36,49], while others focused on alpha and beta frequency bands (7–30 Hz) from frontal electrodes [51] or data provided by NeuroSky [48]. Future studies should investigate whether neural synchrony across different frequency bands or brain regions reveals consistent neural mechanisms. In addition to variations in the brain signal source, there are differences in how the “brain synchrony” index is calculated, which warrants caution when interpreting the results.

Many studies have adopted alpha oscillations as the neural indicator of classroom attention, yet information on other frequency bands has been largely neglected. Ko et al. [53] showed that, in addition to alpha oscillation, beta, theta, and delta oscillations in different brain regions were also associated with longer response times in a sustained visual attention task. Moreover, studies have shown that modulating the power of specific frequency bands, such as sensory motor rhythm (SMR, 12–15 Hz) and beta1 (15–18 Hz or 18–22 Hz), can improve sustained attention in healthy subjects [59,60]. Future studies could further explore the roles of different frequency bands in understanding classroom attention.

### 4.2. Contradictory Findings

Contradictory findings have been observed in studies investigating the same research question. For example, when investigating attention in online learning and classroom learning, while Aggarwal et al. [47] found higher and more sustained attention in the MOOC/e-learning environment, as indicated by the self-train classification model, Horowitz-Kraus et al. [51] found that classroom learning resulted in higher attention, as indicated by greater neural synchrony between the teacher and students, as well as among the students themselves, compared to online learning. Juárez-Varón et al. [52], however, observed similar attention levels between the two modalities.

In terms of the neural differences in classroom activities, while Dikker et al. [37] found that the alpha power was lower during video-watching compared to lectures, Grammer et al. [38] found that the alpha power was higher during video-watching compared to lectures and student-led activities. However, Xu et al. [39] demonstrated that there was no significant difference in alpha power between student-led and teacher-led activities. Potential reasons for these discrepancies could include variations in the neural indicators used or differences in the age groups of participants. For example, developmental studies have documented changes in rhythmic activity in the posterior regions, showing transitions from no measurable posterior basic rhythm in newborns to 4–6 Hz in the first year after birth and progressively increasing to the adult mean of 10 Hz by ages 10–16 [61]. Marcuse et al. [62] also suggest that maturation of the alpha rhythm is not complete until the age of 16. Further research is needed to explore potential reasons for the discrepancies.

### 4.3. Lack of Verification

Stangl et al. [40] argued that, for the cognitive neuroscience field going forward, real-world studies of human cognition are critical to test whether models that have been developed under laboratory conditions hold true in a natural setting. Therefore, it is imperative to include verification from behavior attentional states. However, only 9 out of 16 papers included behavior measurements. We encourage future studies to incorporate behavior measurements, which can bridge findings between laboratory-based experiments and real-world studies, enriching each other with insightful evidence. Addressing the same issue, Janssen et al. [31] propose a cyclical model that integrates three tiers of experimental contexts—lab-based, semi-naturalistic, and fully naturalistic studies—to address complex questions in educational neuroscience. This model emphasizes the complementary strengths of each approach and offers a structured pathway for translating neuroscience findings into authentic educational settings. In lab-based experiments, researchers can tightly control variables and isolate specific neural or cognitive mechanisms, offering high internal validity. These findings can then be tested in semi-naturalistic settings, such as simulated classrooms or one-on-one tutoring scenarios, which introduce greater ecological complexity while maintaining some experimental control. Finally, fully naturalistic studies, conducted in real classrooms with mobile neuroimaging tools, allow researchers to examine how cognitive and neural processes unfold in authentic learning environments, providing high ecological validity. Therefore, we suggest that researchers consider adopting this three-stage framework when investigating issues in educational neuroscience, as it offers a coherent structure for building a complete and logically sound chain of evidence.

### 4.4. Methodological Limitations and EEG Device Heterogeneity

The methodological quality of the included studies presents several limitations that warrant cautious interpretation of findings. One major concern is the significant heterogeneity in EEG devices used across studies, ranging from low-cost, single-channel systems (e.g., NeuroSky, BrainCo Focus 1) to research-grade, high-density equipment (e.g., Brain Products, NeuroScan) with up to 32 channels. This variability directly affects signal fidelity, spatial resolution, and susceptibility to environmental artifacts. Low-density systems, while advantageous for classroom deployment due to ease of use, often yield lower signal-to-noise ratios and limited cortical coverage, which may undermine the validity of derived neural markers such as frequency band power or brain-to-brain synchrony.

In addition, the inconsistent use and reporting of preprocessing pipelines, such as filtering parameters, artifact removal strategies, and feature extraction techniques, further compromise the comparability and reproducibility of findings. Some studies rely on proprietary algorithms (e.g., NeuroSky’s attention meter) with undisclosed methodologies, limiting transparency. These methodological inconsistencies are especially problematic when interpreting neural measures, which are sensitive to channel placement, signal quality, and phase alignment methods.

Taken together, these issues highlight the pressing need for future research to adopt standardized EEG acquisition protocols, transparent preprocessing workflows, and explicit rationale for device selection. Establishing common guidelines will be essential for improving data quality, enabling cross-study comparisons, and enhancing the overall reliability of neural measurements of attention in classroom settings.

### 4.5. Limited Sample Sizes and Techniques

The included studies had limited sample sizes, with an average of around 16 participants and a standard deviation of 8.6, and the smallest sample size being 3 participants. Similar issues were highlighted by Tenório et al. [32] in the context of technology-enhanced learning. According to Schanzenbach [63], small sample sizes may lead researchers to neglect important effects by not rejecting null hypotheses in delicate experiments. Moreover, most of the data collected come from university students, leading to underrepresentation of younger students. As pointed out by Xu et al. [39], differences in the age groups of participants could potentially explain the discrepant findings reported in previous studies. Therefore, future research should consider diverse age groups, adequate sample sizes, and study durations to ensure the reliability and comprehensiveness of the evidence.

Lastly, all of the included studies used EEG to measure neural activities. Although some fNIRS studies have been conducted in an educational context, especially in the field of interpersonal education neuroscience [33,34], fNIRS has been rarely used to measure attention in classroom environments. A notable exception is the study by Brockington et al. [64], which provided proof of concept for using fNIRS to investigate attention in educational settings. In one experiment, the authors demonstrated the feasibility of recording hemodynamic signals from multiple students attending a lecture (without an instructor present) and found significant inter-subject synchronization of oxyhemoglobin signals during the first two minutes of video viewing. In a subsequent experiment, they examined an instructor–student dyad and explored the technical feasibility of simultaneous fNIRS and eye-tracking recordings to assess overt attention. While the study did not report empirical findings on attentional processes per se, it highlights the methodological potential of combining fNIRS with behavioral measures in classroom research.

Functional NIRS can localize brain activity to specific cortical regions (e.g., prefrontal cortex) with reasonable spatial accuracy (typically within centimeters), providing better spatial resolution compared to EEG. Also, EEG recordings are more sensitive to movement artifacts, which can affect data quality, especially in settings involving children or educational tasks that require physical interaction. In contrast, fNIRS is less affected by movement artifacts due to its use of near-infrared light, making it more suitable for studying brain activity in dynamic educational environments such as classrooms. One potential barrier to classroom implementation of fNIRS is the high cost and limited scalability of current fNIRS systems, which makes it impractical to equip entire classrooms for large-scale, multi-subject data collection. However, with the development of more portable and affordable fNIRS systems, or by collecting data from a small subset of students in the classroom, future research can begin to explore the potential of fNIRS for investigating the neural mechanisms of attention in classroom settings.

### 4.6. Future Directions

While our review focused specifically on studies that explicitly examined neural correlates of attention and engagement, other constructs, such as mind wandering, boredom, drowsiness, and flow, represent important dimensions of attentional dynamics in classroom contexts. These states reflect fluctuations in attention and are highly relevant to understanding how students allocate cognitive resources during learning. Future research should explore how these related constructs are represented in neural activity, particularly in naturalistic classroom settings, and how they interact with or diverge from canonical definitions of attention. Integrating these constructs into classroom neuroscience research may offer a more nuanced and comprehensive understanding of learners’ attentional states and their impact on educational outcomes.

Furthermore, the broader field of interpersonal synchrony is rapidly expanding. Contemporary research suggests that synchrony encompasses more than just neural coupling: it includes behavioral (e.g., gaze alignment [14], gesture mimicry [65]) and affective (e.g., shared emotional states [66]) dimensions. For example, Christou et al. [67] employed an eye-tracking paradigm with parent–child dyads and found that gaze congruency in response to emotional expressions was significantly mediated by the child’s aesthetic sensitivity (AES), a subdimension of sensory processing sensitivity. Furthermore, Thompson et al. [68] used EEG hyperscanning to explore parent–child interpersonal neural synchrony (INS) and reported that higher levels of INS were positively correlated with family functioning and relational warmth. Their findings emphasize that synchrony is not only a reflection of shared attention, but also a relational marker embedded in affective and social bonds. Future educational neuroscience research may benefit from integrating EEG with other methods such as eye tracking, motion capture, physiological monitoring, or observational behavioral coding to provide a more ecologically valid and holistic view of how attention and engagement unfold in socially embedded learning environments.

## Figures and Tables

**Table 1:** Criteria for inclusion and exclusion in this scoping review.

Criterion	Inclusion	Exclusion
Article type	Original research, including peer-reviewed journal articles, dissertations, and conference papers	Articles that were not original research (e.g., literature reviews, meta-analyses, editorials, book reviews, or reports)
Literature focus	Investigate the neural activity/neural mechanism/brain signal of attention/engagement in face-to-face classroom settings	Behavioral studies, pure online learning/e-learning studies, research topics unrelated to attention
Language	English	Non-English
Publication date	2001–2024	Studies outside this period
Age group	All age groups	-
Population type	Healthy participants	Participants with neural conditions (e.g., ADHD)
Education level	Kindergarten, K–12, higher education, and professional training	-
Disciplinary field	All fields	-

**Table 2:** Data coding and extraction form.

Characteristics	Information	Relevant RQ
Author	Name of the first author	Publication characteristics
Publication year	The time when the article was published	Publication characteristics
Region	The place where the research was conducted	Publication characteristics
Publication type	Peer-reviewed journal article, dissertation, or conference paper	Publication characteristics
Objectives	Purpose of the selected study	RQ1
Outcomes	Outcomes of the selected study	RQ1
Technique and device	Electroencephalogram (EEG), functional near-infrared spectroscopy (fNIRS), or other techniques and their devices	RQ2
Neural measurement of attention	Neural indicator to measure attention level	RQ3
Behavioral measurement of attention	Whether included behavioral measurement of attention. If yes, how?	RQ3
Classroom activities	Lectures, videos, group discussion, polling, etc. Neural correlates of attention during classroom activities	RQ4
Education level	Kindergarten, K–12, higher education, or professional training	RQ5
Disciplinary field	Disciplinary fields	RQ5
Sample size	Sample sizes of neural data collected	RQ5
Duration	Number of sessions and duration of each session	RQ5

**Table 3:** Information about the included papers.

No	Authors	Year	Publication Type	Technique	Device	Channel
1	Aggarwal et al. [47]	2021	Journal paper	EEG	Neurosky Mindwave	1
2	Bevilacqua et al. [36]	2019	Journal paper	EEG	Emotiv EPOC	14 *
3	Chen et al. [48]	2024	Journal paper	EEG	NeuroSky	1
4	Davidesco et al. [49]	2023	Journal paper	EEG	Neuroelectrics	32
5	Dhindsa et al. [50]	2019	Journal paper	EEG	Not reported	16
6	Dikker et al. [29]	2017	Journal paper	EEG	Emotiv EPOC	14 *
7	Dikker et al. [37]	2020	Journal paper	EEG	Emotiv EPOC	14
8	Grammer et al. [38]	2021	Journal paper	EEG	SMARTING mobile EEG	24
9	Horowitz-Kraus et al. [51]	2023	Journal paper	EEG	Not reported	Not reported
10	Juárez-Varón et al. [52]	2023	Journal paper	EEG	Emotiv EPOC	14
11	Ko et al. [53]	2017	Journal paper	EEG	NeuroScan	32
12	Kosmyna et al. [54]	2019	Journal paper	EEG	BrainCo Focus 1	1
13	Sun [55]	2014	Journal paper	EEG	Not reported	Not reported
14	Sun, Chen et al. [56]	2018	Journal paper	EEG	NeuroSky	1
15	Sun, Hwang, et al. [57]	2018	Journal paper	EEG	NeuroSky	1
16	Xu et al. [39]	2022	Journal paper	EEG	Study 2: SMARTING mobile EEG	24

* Data from six channels were used.

**Table 4:** Information about classroom activities.

No.	Author	Classroom Activities	Neural Activities for Different Classroom Activities
2	Bevilacqua et al. [36]	Videos and lectures	Student-to-group synchrony, student–teacher synchrony, and engagement were all higher for videos as compared with lectures.
6	Dikker et al. (2017) [29]	Videos, lectures, and group discussions, and the teacher reading aloud	Student-to-group synchrony was higher for video and group discussions than lecture sessions.
7	Dikker et al. (2020) [37]	School 1: Videos, lectures, group discussions, and the teacher reading aloud; School 2: Videos and lectures	Lower alpha power during videos compared to lectures. Theta power did not vary by class activity.
8	Grammer et al. [38]	Teacher-led activities (videos and lectures) and student-led activities (group work and independent work)	Higher alpha power over the occipital cortex during video watching compared to lectures and student-led activities. Lower alpha, higher beta, and higher gamma power during student-led activities than teacher-led activities.
13	Sun [55]	Lectures and polling (clickers and mobile polling)	Students’ brainwave data related to attention increased during polling activities as opposed to the lecture in general.
14	Sun, Chen et al. [56]	Lectures, polling, concept mapping drawing, votable concept mapping	The attentional neural activities of the three students varied across different types of instructional methods and activities.
15	Sun, Hwang, et al. [57]	Lectures and polling (clickers, group polling, group polling with competition)	The attentional neural activities of the three students varied across different types of instructional methods and activities.
16	Xu et al. [39]	Mindfulness, teacher-led activities (lecture), and student-led activities (card games played in pairs and crafting activities done individually)	Alpha power was highest during mindfulness. No significant difference between teacher-led and student-led activities.

**Table 5:** Information about the neural and behavioral measurements used in the included studies.

No.	Author	Neural Measurement	Behavioral Measurement
1	Aggarwal et al. [47]	Self-trained classification model based on eight frequency bands (high α, mid α, low α, high β, low β, γ, θ, and δ) of the EEG signal and R1: (low α/low β), R2: (high α/ high β)	Students’ self-feedback about their state of mind as seen in the video and other distractions like losing eye contact with the teacher or teaching board were also considered
2	Bevilacqua et al. [36]	Brain-to-brain synchrony (student-to-group synchrony, student–teacher synchrony) at F3, F4, P7, P8, O1, and O2 sites	1–7 Likert engagement rating by students
3	Chen et al. [48]	Brain-to-brain synchrony (student-to-group synchrony, based on attention level provided by NeuroSky)	-
4	Davidesco et al. [49]	Brain-to-brain synchrony (based on phase) at center, frontal, and posterior electrodes	-
5	Dhindsa et al. [50]	Self-trained classification model based on four frequency (θ, α, β1, βa2) bands of the EEG signal	Thought probes
6	Dikker et al. (2017) [29]	Brain-to-brain synchrony (student-to-group synchrony) at F3, F4, P7, P8, O1, and O2 sites	1–7 Likert engagement rating by students
7	Dikker et al. (2020) [37]	α power (highest local maximum) at all occipital electrodes	Self-reported focus scores
8	Grammer et al. [38]	α power on Pz, POz, O1, and O2 electrodes (β, θ, δ power reported in the supplementary information)	Observer ratings based on behavioral cues, including body positioning, eye gaze, and activity engagement in 1 min intervals
9	Horowitz-Kraus et al. [51]	Brain-to-brain synchrony (teacher-students synchrony) at α and β frequency at frontal electrodes (AF3, AF4, F4, F3, F8, and F7)	-
10	Juárez-Varón et al. [52]	Not reported	-
11	Ko et al. [53]	α, β, θ, δ power	Sustained attention task
12	Kosmyna et al. [54]	Index E = β/(α + θ)	Self-report engagement score
13	Sun [55]	Not reported	-
14	Sun, Chen et al. [56]	Attention level provided by NeuroSky	Three observers coded the attentional behavior of the participants
15	Sun, Hwang, et al. [57]	Attention level provided by NeuroSky	-
16	Xu et al. [39]	α power on Pz, POz, O1, and O2 electrodes (β, θ, δ power reported in the supplementary information)	-

**Table 6:** Information about the educational levels, disciplinary domains, durations, and sample sizes of the included studies.

No.	Authors	Education Level	Disciplinary Domain	Duration	Sample Size
1	Aggarwal et al. [47]	Higher education	Machine learning	1 session of 15–20 min	12
2	Bevilacqua et al. [36]	High school	Biology	6 sessions of 80 min	12
3	Chen et al. [48]	Primary school	Brain sciences	5 sessions of 20 min	24
4	Davidesco et al. [49]	High school and higher education	Biology and Chemistry	4 sessions of 7-min	31
5	Dhindsa et al. [50]	Higher education	Medicine	2 sessions of 30 min	15
6	Dikker et al. [29]	High school	Biology	11 sessions of 50 min	12
7	Dikker et al. [37]	High school	Biology	School 1: same as Study 5; School 2: same as Study 2	22
8	Grammer et al. [38]	Higher education	Educational neuroscience	1 session of 40–60 min	23
9	Horowitz-Kraus et al. [51]	Primary school	Science	2 sessions of 15 min	6
10	Juárez-Varón et al. [52]	Higher education	Consumer behavior	5 sessions of 45 min	20
11	Ko et al. [53]	Higher education	Not reported	2–8 sessions of 50 min (mean 5.4 sessions)	18
12	Kosmyna et al. [54]	Higher education	VR	3 sessions of 40–50 min	12
13	Sun [55]	Higher education	Educational research methods and sociology of education	16 sessions	32
14	Sun, Chen et al. [56]	Higher education	Educational research methodology	3 sessions of 100 min	3
15	Sun, Hwang, et al. [57]	Higher education	Not reported	6 sessions of 100 min	3
16	Xu et al. [39]	kindergarten to 4th-grade students	Neuroscience	3 sessions of 24 min	10
